# SITbench 1.0: A Novel Switch-Based Interaction Technique Benchmark

**DOI:** 10.1155/2019/5075163

**Published:** 2019-08-18

**Authors:** Cagdas Esiyok, Sahin Albayrak

**Affiliations:** Distributed Artificial Intelligence Laboratory, Technische Universität Berlin, Berlin, Germany

## Abstract

Evaluation process of a switch-based interaction technique (SIT) requires an interdisciplinary team effort and takes a considerable amount of time. Collecting subjective evaluation data from the users is a very common approach, but the subjective evaluation data alone might be manipulated and unreliable for comparing performances in many cases. Thus, therapists generally cannot succeed in determining the optimum SIT setup (i.e., determining the most appropriate combination of setup variables such as the switch type or switch site) at first attempts since it is hard to evaluate the measurable performance by collecting subjective data instead of objective data. Inevitably, each unsuccessful attempt to reach the optimum SIT setup results in a loss of serious time and effort. On the contrary, a benchmark application is also required to make performance evaluation of SITs by using a number of standard tests and empirical attributes. It is obvious that a quicker and more accurate SIT evaluation process provides a better cost and schedule management considering the increasing number of SIT users in the world. Therefore, we propose a novel benchmark for performance evaluation called SITbench that provides a quicker and more accurate switch evaluation process by collecting and saving the objective data automatically. We conducted a user study with eight participants and demonstrated that the objective data collected via the SITbench helped to determine the optimum SIT setup accurately. Result of a questionnaire applied to evaluate the SITbench itself was also satisfactory. SITbench is expected to help researchers and therapists to make a better evaluation according to any change done in SIT setup variables (switch type, activation method, etc.) with the aim of reaching the optimum SIT setup, which leads to a better cost and schedule management. As the first benchmark application compatible with all SITs, which can emulate keyboard characters or mouse clicks, it can be utilized by assistive technology professionals to make comparisons and evaluations automatically via standardized tests.

## 1. Introduction

There have been many people with motor disabilities worldwide [1] who depend on SITs for communication or any other reasons. SITs are one of the most important assistive technology solutions for people with motor disabilities. Therefore, researchers and manufacturers developed plenty of different SITs which can be separated into two main groups as traditional hardware switches and virtual switches: while traditional switches are electronic devices varying from breath-activated ones [2] to tooth-click switches [3], virtual switches [4–6] are generally computer applications that imitate switch functions in a way that a specific body gesture detected via sensors or input devices (such as head nodding detected by a camera) is considered as an activated switch. They assist users to interact with their environment. For example, a user can select a target on the computer screen by hitting a switch [7] or an electric wheelchair can be operated via multiple switches [8]. Evaluation process of a SIT is the most important task in order to determine the optimum SIT setup for motor-impaired people.

It is obvious that an efficient evaluation process of a SIT helps therapists to determine the optimum SIT setup for motor-impaired people, but there are many variables in a SIT setup such as switch type, switch site, users' posture, and activation method. For example, even a button switch can be used in several ways: it can be activated by the hand or any other body part. Likewise, users can be positioned in different postures during switch usage, which might affect the performance dramatically. The main aim of a SIT evaluation is to determine the optimum SIT setup which is the most suitable combination of these variables for the users to interact with their environment. To this end, a considerable time and effort is needed by an interdisciplinary team that includes many trials with different variables of SIT setup. On the contrary, assistive technology professionals require a benchmark application [9], which is compatible with most SITs, to make a better comparison and evaluation automatically with standardized tests under the same conditions. Considering the increasing number of SIT users, any tool that allows a more accurate and quicker SIT evaluation process becomes an important requirement day by day.

Currently, SIT evaluation is performed in three ways: (a) collecting subjective data [5, 10–13] via questionnaires, observations, and interviews by an interdisciplinary team; (b) collecting objective data [3, 6, 14–20] via performance tests; and (c) collecting both subjective and objective data [4, 21, 22]. Because the subjective data alone might be unreliable and manipulated easily for performance evaluation, it might be hard to succeed in determining the optimum switch setup on the first attempts in many cases by therapists. They might need several attempts by reapplying questionnaires or making new observations. For each unsuccessful attempt, serious time and effort are required to collect a new subjective data. Thus, collecting subjective data is not a proper way to evaluate the measurable performance of a SIT. Without a performance evaluation, it might be very challenging to achieve the optimum SIT setup with subjective evaluation alone. On the contrary, although collecting objective data is the most appropriate method for performance evaluation, current objective evaluation methods in literature are far from being a benchmark. These methods are mostly designed to evaluate just a specific SIT, which makes them ineligible to be a benchmark where the other SITs could be evaluated via a standardized test. To the best of our knowledge, there are only two evaluation applications in literature [19, 20] which are close to be a benchmark for SIT evaluation. They can provide quantitative data to evaluate computer access skills and help therapists to choose the switch type and position, but both applications have some common limitations that we aim to overcome with our novel SITbench:

1. Incompatibility: switch-accessible applications might require different keyboard characters or mouse clicks from switches to work. Furthermore, each switch might emulate and send different keyboard characters or mouse clicks depending on its manufacturer. Unfortunately, commonly agreed standard is not available. For example, while some switch-accessible applications might expect to receive a keyboard space character, other applications might expect to receive a mouse right-click. Both applications expect to receive a mouse left-click to work. In other words, they are only compatible with switches which are able to emulate mouse left-click. The remaining switches are excluded, which means that just a minority of SITs are compatible and could be evaluated with these applications. Therefore, we consider that they are far from being a proper benchmark for SIT evaluation. Our novel tool SITbench is compatible with all switches, which can emulate any mouse clicks or keyboard characters, since it allows therapists to assign the expected characters from any switch.
2. Limited number of switches: they are only capable to evaluate single-switch systems. Double-switch support is also required since double-switch usage is widely used as an alternative interaction method. SITbench is capable to allow both single- and double-switch evaluation.
3. Limited number of tests: both applications employ only one test that measures press time (i.e., the time from the prompt to when the switch is pressed) and release time (i.e., the time from when the switch is pressed until it is released) of a switch. SITbench includes two more additional tests to evaluate SITs with the single and double switch.
4. Database requirement: they have some reporting functions for the test results. However, we considered that a well-structured database would be useful to share the results and apply some queries or statistical tests. In addition to the reporting function, SITbench also allows to save the test results automatically into a Microsoft Access database.
5. Sufficient attention span requirement: sufficient attention span via both applications might not be achieved especially by infants since they can become distracted and lose their attention easily during long and boring sessions. We applied gamification techniques while designing SITbench tests with the intent to make evaluations more engaging and fun.

Therefore, we propose a novel SIT evaluation tool, namely, SITbench as a benchmark application which helps to determine the optimum SIT setup with the aim of providing a quicker and more accurate SIT evaluation process. To collect the objective data, SITbench includes three different games which can be played via the single or double switch. It measures and saves the performance metrics (accuracy, precision, recall, and false-positive rate) automatically at the end of each trial.

A user study with eight participants was conducted as a part of this work in order to test and demonstrate the proposed benchmark application. We identified two different switch sites to be tested by users under the same conditions in order to determine the most suitable switch site. To this end, we collected the objective data via SITbench. Results revealed that SITbench could help to determine the optimum switch setup accurately. We also applied a System Usability Scale (SUS) [23] questionnaire to evaluate the SITbench itself, and the results were quite satisfactory.

More potential SIT users can be served at the same time period with the same workforce since a quicker and more accurate SIT evaluation process is provided by the SITbench, which might prevent governments to spend high amounts of money as a result of better cost and schedule management. As a benchmark application, it allows to make objective comparisons with standardized tests under the same conditions by collecting the performance data of SITs for assistive technology community automatically. Thus, it provides extratime for therapists to observe more subjective aspects of client needs. On the contrary, it might be used to evaluate fine-motor skills of clients as a clinical tool. Occupational therapists can track the patients' progress by SITbench that allows to measure and record clients' fine-motor performance and reflexes automatically in the form of quantitative objective data. The SITbench might also help to improve the contingency awareness of the ones with profound and multiple learning disabilities, or it might be useful for pupils with severe learning difficulties to assess their auditory and visual attention.

This paper begins with the section that presents the design and implementation of our novel switch evaluation tool. Then, in Evaluation, we share the objective results of our user study and the questionnaire results of the SITbench. Finally, we conclude our study and discuss our future work in Conclusion.

## 2. SITbench Design

**Figure 1:**
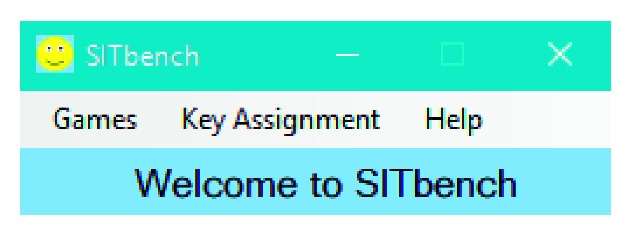
Welcoming screen of SITbench.

SITbench is designed as a novel benchmark application for assistive technology and healthcare professionals to determine the most appropriate SIT setup. It helps to collect and save the objective data automatically with the aim of the optimum SIT setup. To this end, three different switch-accessible games, depending on the single or double switch, were designed within SITbench, namely, *Tie-Smiley Matching Game*, *Nonstop Driver Game*, and *Hungry Frog Game*, respectively. SITbench welcomes users with a very simple interface (Figure 1) when it is initialized. In the welcoming screen, users can select the games (i.e., tests) and open the key assignment module to assign the expected keys from switches.

**Figure 2:**
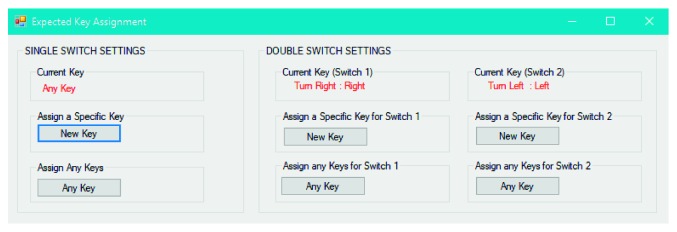
Expected key assignment module.

As can be seen in Figure 2, single- and double-switch settings can be configured according to the expected key (i.e., a keyboard character or a mouse click) from any SITs to be tested via SITbench. In this way, SITbench becomes compatible with the majority of assistive switches since almost all switches on the market can emulate a keyboard character or a mouse click.

### 2.1. Tie-Smiley Matching Game (TSMG)

**Figure 3:**
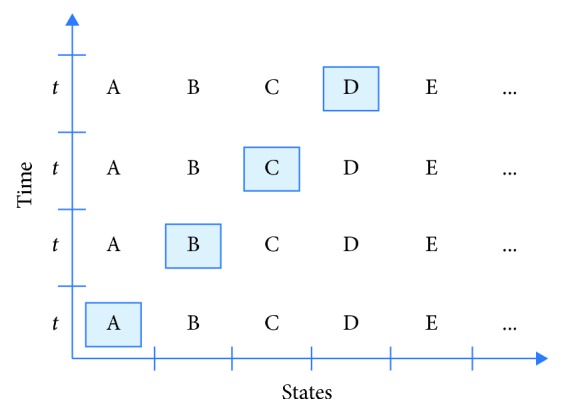
Time-state model of an automatic linear scanning sample.

TSMG is a single switch-accessible game based on indirect selection with the automatic linear scanning method. As it is exemplified in Figure 3, an indirect selection with the automatic linear scanning method can be summarized in three steps: (1) letters in a scanning array (English alphabets as a selection set) are highlighted one-by-one on the screen for an equal duration (*t* units of time where *t* represents scan time, i.e., the time interval between two successive states); (2) until the end of each state, the user is allowed to make a selection by hitting a switch or sending any kind of signal detected by a sensor (e.g., a blink); (3) if the highlighted letter is the target (i.e., what user intends to select), the user sends a selection signal such as blinking.

**Figure 4:**
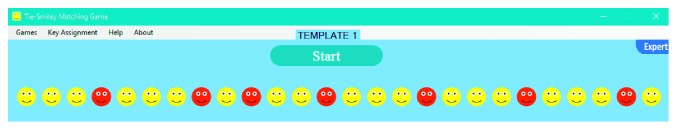
Initial form of TSMG in template 1.

There are five different templates which could be tested via TSMG.  Figure 4 shows an initial form of template 1. The scanning array of each template consists of yellow and red smileys (26 smileys in total). Red smileys are targets to be selected, and they are set in a different order for each template in order to avoid repetition. Targets are seen by user before starting and during the test.

**Figure 5:**
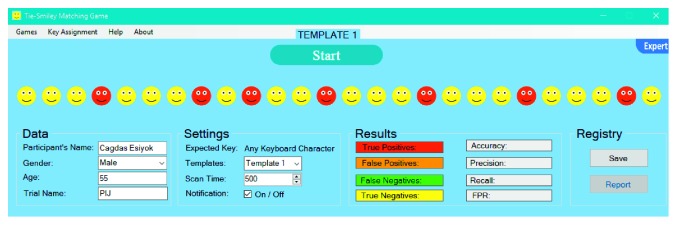
Initial form of TSMG in the expert mode.

TSMG is based on automatic linear scanning where each smiley is highlighted for a given time period (i.e., scan time) one-by-one. User should activate the switch once the highlighted smiley is a red one. User also hears a click sound as an auditory prompt, as soon as the target is highlighted. When the switch is activated, it sends the expected key to SITbench as a selection signal. Once the expected key is received, SITbench gives a sensory feedback by swapping the background color of the interface like a blink. In the expert mode (Figure 5), therapists can enter some details about the user. They can also select the template and set the scan time (in milliseconds).

**Figure 6:**
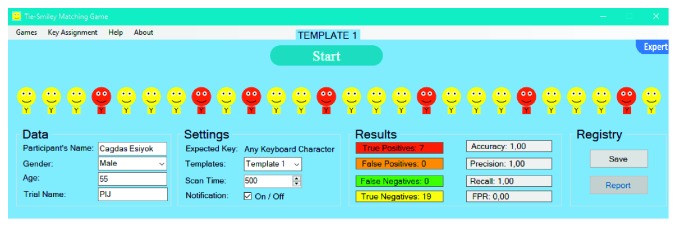
A view of TSMG in the end of trial following a user performance without any mistake.

User aims to match each smiley with a tie in a way that smiley and its tie are in the same color (e.g., red smileys with red ties). To this end, the user should select all red smileys but yellow ones via a switch. A sample view of results after the user completed a trial without any mistake can be seen in Figure 6.

**Figure 7:**
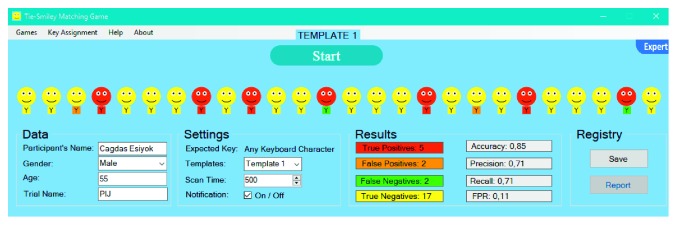
A general view from TSMG in the end of trial following a user performance with several mistakes (i.e., with false negatives and false positives).

At the end of each trial, confusion matrix variables (true positives (TP), false positives (FP), false negatives (FN), and true negatives (TN)) are calculated and assigned automatically, as can be seen in Figure 7, according to the count and color of ties in a way that TP represents the count of red ties, FP represents the count of orange ties, FN represents the count of green ties, TN represents the count of yellow ties. All performance evaluation metrics (accuracy, precision, recall, and false-positive rate) are measured by SITbench automatically at the end of trial by using the following formulas:

(1) $$\begin{array}{c} \text{accuracy}=\frac{\text{TP}+\text{TN}}{\text{TP}+\text{TN}+\text{FP}+\text{FN}},\\ \text{precision}=\frac{\text{TP}}{\text{TP}+\text{FP}},\\ \text{recall}=\frac{\text{TP}}{\text{TP}+\text{FN}},\\ \text{false}-\text{positive rate}=\frac{\text{FP}}{\text{FP}+\text{TN}}. \end{array}$$

SITbench allows therapists to save the results and all data into a structured database, and it has also a reporting function to print or save the results as a document.

### 2.2. Nonstop Driver Game (NDG)

**Figure 8:**
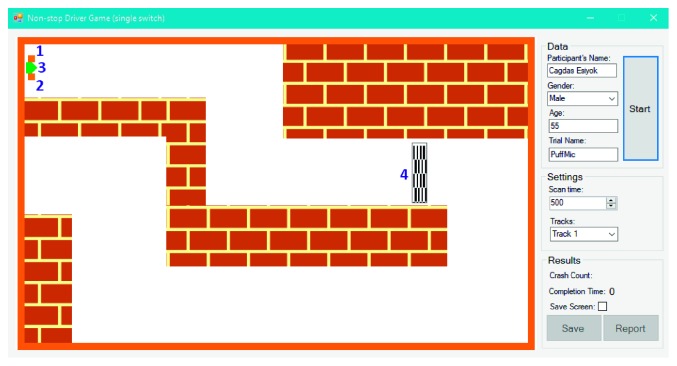
The initial form of SS-NDG where 1 shows the left signal (i.e., orange square box), 2 is the right signal; 3 represents the green car, and 4 is the finish line.

**Figure 9:**
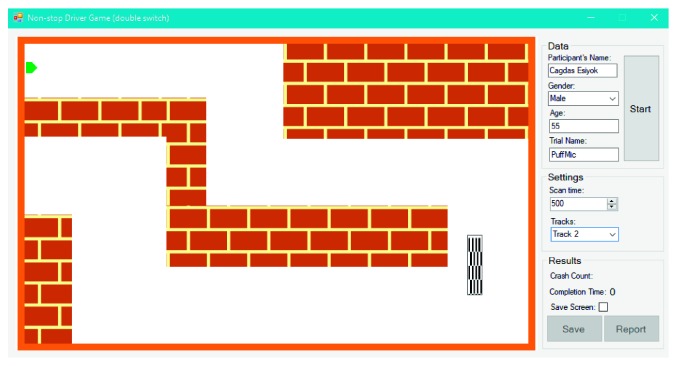
The initial form of DS-NDG in track 2.

**Figure 10:**
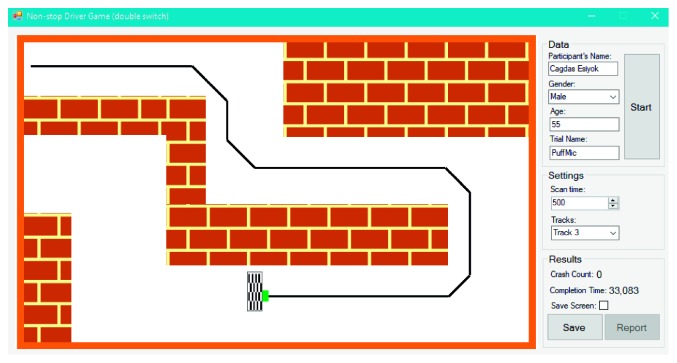
A view of DS-NDG in the end of trial where the user reached the finish line in track 3 without any crash.

NDG can be played with the single or double switch: (1) a single switch-accessible version based on indirect selection (SS-NDG); (2) a double switch-accessible version based on direct selection (DS-NDG).  Figure 8 shows the initial form of SS-NDG. Game objects are labeled with blue numbers in Figure 8 to introduce them: label 1 shows the left signal (i.e., orange square box); 2 is the right signal; 3 represents the green car; 4 is the finish line. Before starting the game, therapists can select the track and adjust the scan time. The aim of the user is to reach the finish line as soon as possible with minimum crash into the walls. SS-NDG depends on the automatic scanning method with a single switch where signals (i.e., car's left and right signals which are illustrated as orange boxes) are flashed one by one for a given time period (i.e., scan time in milliseconds). After the game starts, the car begins to move and never stop until reaching the finish line. To turn the car left, the user hits the switch once the car's left signal is flashed and hits the switch once the right signal is flashed to turn the car right. The only difference between SS-NDG and DS-NDG is that the car in DS-NDG has no left and right signals (Figure 9) since it is controlled with a double switch. User activates the first switch to turn it left and the second switch to turn it right. We also assigned two different sounds to SITbench as auditory prompts according to left and right signals. In other words, the user hears two different sounds when signals are flashed during the game. Once the user hits the switch, the expected key is received and SITbench provides a sensory feedback visually by swapping the background color of the interface. There have been five different tracks where each track has a different finish line location from each other to avoid the learning effect. A sample view in the end of trial is shown in Figure 10 where the user completed the game via the double switch in track 3 without any crash. Completion time (in seconds) and crash count are measured automatically as performance metrics at the end of each trial. SITbench enables therapists to save all results and data into a database and to report them as a document. It also depicts a black tracking line of the car (Figure 10) and allows therapists to save the print screen of the interface as a separate image file.

### 2.3. Hungry Frog Game (HFG)

**Figure 11:**
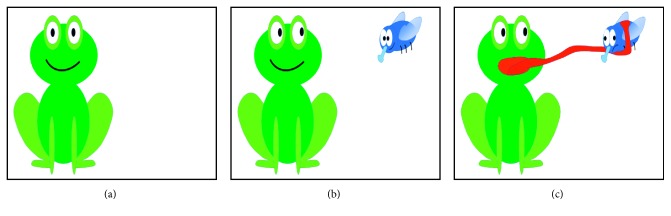
All three frames shown to user during a task: (a) frame shown until a fly is appeared; (b) frame shown until the user activates the switch; (c) frame shown once the user activates the switch.

**Figure 12:**
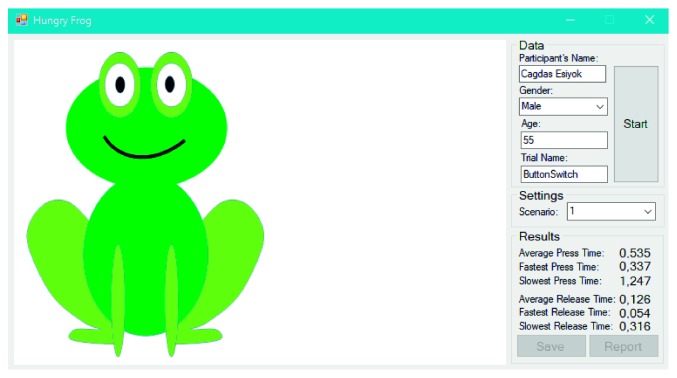
A view of HFG in the end of a trial.

HFG is a single switch-accessible application to measure user's switch performance. At the beginning of each trial, therapists can select the scenario and enter user details. Each trial of game consists of ten tasks. As it is illustrated in Figure 11, each task in a trial is achieved in a way that (a) the user waits until a fly is appeared, (b) the user activates the switch as soon as fly is seen, and (c) frog eats the fly once the user activates the switch. As soon as the fly appears, the user hears a click sound as an auditory prompt. When the expected key is received from the switch, the background color of SITbench is swapped like a blink to give a sensory feedback. After the user completes ten tasks, SITbench measures average press time (i.e., the average time from when the fly appears to when the switch is pressed) and average release time (i.e., the average time from when the switch is pressed until it is released) automatically. The fastest and the slowest press time and release time among ten tasks are also detected. HFG has five different scenarios to avoid repetition and learning effect. For each scenario, waiting times (i.e., the time from when the user starts to wait to when the fly appears and not more than 6 seconds) of each task in a trial are set different from each other.  Figure 12 shows the view of interface in the end of each trial. Six performance metrics (measured in seconds) can be saved into a database and reported via SITbench: (1) average press time, (2) average release time, (3) the fastest press time, (4) the slowest press time, (5) the fastest release time, and (6) the slowest release time.

## 3. Evaluation

**Figure 13:**
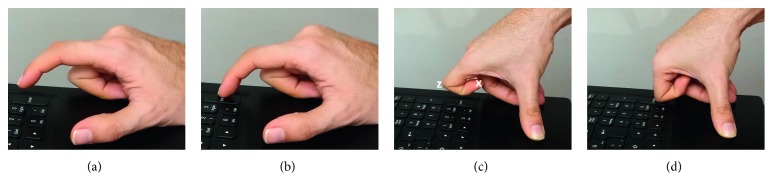
Positions of forefinger during experiments according to two switch sites FDP (represented by *x*) and FPIJ (represented by *z*): (a) switch press with FDP; (b) switch release with FDP; (c) switch press with FPIJ; (d) switch release with FPIJ.

We conducted a user study as a demonstration of SITbench. We identified two different switch sites (Figure 13(c)) to be tested: forefinger distal pulp (FDP) and forefinger proximal interphalangeal joint (FPIJ). FPD was considered as a proper switch site to activate a switch easily in contrast to FPIJ. We aimed to demonstrate that SITbench can determine the most proper switch site. To this end, users performed tests by using two different switch sites. A questionnaire was also applied to evaluate SITbench itself.

In this section, firstly we introduce the participants. Then, we present the apparatus used and the procedure applied. At last, we share the experimental findings.

### 3.1. Participants

Eight able-bodied participants (mean age = 30.2, standard deviation = 3.1), including four females and four males, took part in this study. Just two of the participants were familiar with switch-accessible applications before experiments.

### 3.2. Apparatus

A laptop (model: Lenovo G505S; CPU: AMD A8-4500M 1.9 GHz; RAM: 6 GB DDR3; screen: LCD 15.6; OS: Windows 10 64 bits; resolution: 1600 × 900) was employed within this study for experiments.

### 3.3. Procedure

At the beginning, the participant is positioned in front of a laptop in a way that the participant is able to access laptop's keyboard easily. *Enter* key on the keyboard was considered as a switch.

Participants were informed about the SITbench and tests, and then they practised SITbench in the counterbalanced order until they become ready for tests. This practicing step took 20 minutes approximately for each participant. Following positioning and practicing steps, three tests were applied to participants to collect objective performance data:

1. TSMG: each switch site (FDP and FPIJ) was tested by each participant (*n* = 8) for each template (*n* = 5) two times where scan time is 500 milliseconds (i.e., each participant performed 20 trials in total with TSMG).
2. SS-NDG: each switch site (FDP and FPIJ) was tested by each participant (*n* = 8) for each track (*n* = 5) where scan time is 500 milliseconds (i.e., each participant performed 10 trials in total with SS-NDG).
3. HFG: each switch site (FDP and FPIJ) was tested by each participant (*n* = 8) for each scenario (*n* = 5) (i.e., each participant performed 10 trials in total with HFG).

All tests were applied in the counterbalanced order to avoid learning and repetition effects. The participants were also allowed to rest (1 to 5 minutes) during experiments to prevent excessive mental or physical fatigue.

At the end of experiments, a questionnaire based on SUS [23], which is an industry standard, was applied to the participants to evaluate the usability of SITbench application. SUS includes ten statements (Table 1) with a five-point Likert scale. Scale value of statements is ranging from 1 (strongly disagree) to 5 (strongly agree). We modified SUS statements according to SITbench to clearly describe it. The SUS score is calculated as follows: (1) sum the score contributions of each statement (ranging from 0 to 4) where for statements 1, 3, 5, 7, and 9, the score contribution is the scale value minus 1; for statements 2, 4, 6, 8, and 10, the score contribution is 5 minus the scale value; (2) multiply the sum of the score contributions by 2.5 to get the SUS score (ranging from 0 to 100). On the contrary, qualitative subjective data were collected via our observations and participants' responses of open-ended questions about SITbench.

### 3.4. Results

#### 3.4.1. Subjective Results

Results of the SUS questionnaire are listed in Table 1. The scale column holds the average scale values (1 to 5) of each statement for all the participants. The average SUS score for all the participants was calculated as 84 (minimum score = 70, maximum score = 95, and standard deviation = 8.4). According to the adjective rating scale [24], the overall SUS score (84) of SITbench was rated as excellent, and SUS scores of each participants ranged from good to excellent. Prior to experiments, all the participants were excited for experiments. Just two of them had a previous experience with SITs. They all declared that SITbench would be a very useful tool for assistive technology community. One participant stated that he could have performed better if scan time was slower. Two of the participants suggested to increase the size of smileys in TSMG. All the participants declared that FDP is definitely more proper than FPIJ as a switch site. None of the participants experienced fatigue during tests.

#### 3.4.2. Objective Results

FDP as a switch site showed quite impressive performance in comparison with FPIJ in all three tests (TMSG, SS-NDG, and HFG) as it is expected at the beginning. It is demonstrated that SITbench succeeded to determine the most appropriate switch site as FDP.

**Figure 14:**
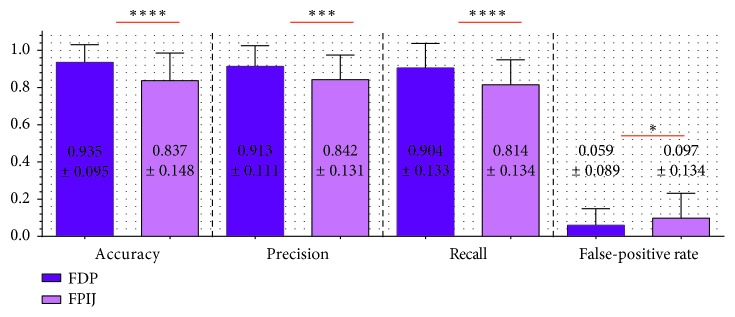
Mean values and standard deviations of two switch sites for all the participants through evaluation metrics of TSMG (accuracy, precision, recall, and false-positive rate) (^*∗*^*p* value < 0.05; ^*∗∗∗*^*p* value < 0.001; ^*∗∗∗∗*^*p* value < 0.0001).

According to results of TSMG (Figure 14), FDP was better than FPIJ in all performance evaluation metrics (accuracy, precision, recall, and false-positive rate).

**Figure 15:**
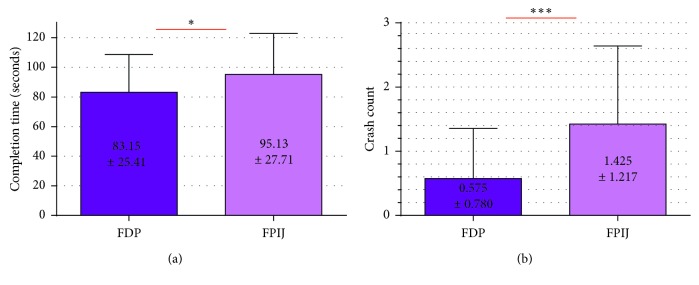
Mean values and standard deviations of two switch sites for all the participants according to evaluation metrics of SS-NGD as (a) completion time and (b) crash count (^*∗*^*p* value < 0.05; ^*∗∗∗*^*p* value < 0.001).

SS-NDG results (Figure 15) also suggested that FDP performed better than FPIJ in terms of completion time and crash count.

**Figure 16:**
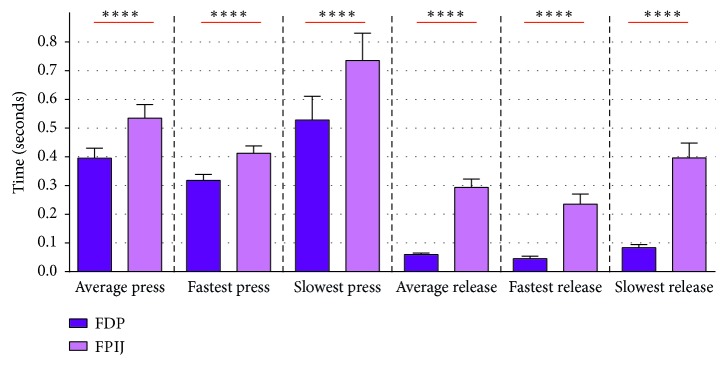
Mean values and standard deviations of two switch sites for all the participants through evaluation metrics of HFG (average press time, average release time, the fastest press time, the slowest press time, the fastest release time, and the slowest release time) (^*∗∗∗∗*^*p* value < 0.0001).

Lastly, HFG results (Figure 16) proved that FDP is by far the best switch site in all evaluation metrics (average press time, average release time, the fastest press time, the slowest press time, the fastest release time, and the slowest release time).

On the contrary, we applied *t*-tests for both switch sites through all evaluation metrics in all three tests. In consequence of *t*-tests, it is proved that there is a significant difference between the performance of FDP and FPIJ for all evaluation metrics.

## 4. Conclusion

Evaluation process is one of the most important tasks in order to reach the optimum SIT setup. Because the optimum SIT setup plays a vital role for people with motor disabilities to interact with their environment, any tool to achieve the optimum SIT setup for having a better cost and schedule management becomes a very important requirement considering the increasing number of SIT users. Determining the optimum switch setup by collecting the subjective data might be challenging since the subjective data alone might be unreliable and manipulated easily for performance evaluation. Therapists might have to reapply questionnaires and make new observations several times. A serious time and effort is needed for these repeated trials to collect subjective data. Therefore, subjective data collection instead of objective data does not seem a proper method for performance evaluation of a SIT. On the contrary, current evaluation methods based on collecting objective data in literature are far from being a benchmark. These methods are generally employed to evaluate just a specific SIT. In other words, they are not designed to evaluate the other SITs, which make them ineligible to be a benchmark. To the best of our knowledge, there have been just two applications [19, 20] in literature which are close to be a benchmark. The main limitations of these applications and solutions we proposed with SITbench are as follows: (a) They only work with the SITs that can emulate mouse left-click, which makes them compatible with just a minority of SITs for evaluation. SITbench as a benchmark allows to assign any expected characters or mouse clicks from any SIT. By this way, all SITs which can emulate keyboard characters or mouse clicks could be evaluated and compared via SITbench with standardized tests. (b) They only support single switch-based systems. Because double-switch usage is a widely preferred interaction technique, SITbench supports double-switch evaluation as well. (c) They have only one test to measure press time and release time of a switch. SITbench has two more performance tests to evaluate SITs. (d) They do not allow to save the results into an external database although they have some reporting functions. SITbench supports to save the result automatically into a database to share it or analyze it for further studies. Therefore, we propose SITbench as a benchmark application that helps to determine the optimum SIT setup to provide a quicker and more accurate SIT evaluation process by collecting and saving the objective data automatically.

We have conducted a user study as a demonstration with eight participants to evaluate the usage of different switch sites. To this end, objective data were collected via SITbench. FDP performed better performance than FPIJ in all tests as it is expected. Findings demonstrated that SITbench is capable to determine the most proper switch site with the aim of an optimum SIT setup. Result of a SUS questionnaire to evaluate the SITbench itself was also quite satisfactory.

A quicker and more accurate SIT evaluation via SITbench helps to serve more potential SIT users at the same time period with the same workforce. As a result of better cost and schedule management, SITbench might prevent governments from unnecessary expenses and human-resource allocations, but future studies with SITbench are required to verify that SITbench is capable to do this. On the contrary, it might be also employed by therapists and assistive technology professionals to measure the fine-motor skills and reflexes of users as a clinical tool. They can track the progress of user's skill via SITbench since it is capable to measure and save the performance automatically as a quantitative objective data. SITbench can also be utilized to improve the contingency awareness of the ones with profound and multiple learning disabilities. Besides, it might be employed as a tool to assess auditory and visual attention of people with severe learning difficulties.

In order to improve the SITbench and overcome some of its limitations, some future studies would be quite useful. We intend to include new tests depending on several scanning methods. So as to test the efficiency of SITbench better, we aim to extend the participant group with motor-impaired people. Since the SITbench is currently compatible with only desktop computers, it might be modified to be compatible with mobile systems such as smartphones and tablets to extend the target group. We also aim to include some tests such as a speller to evaluate users' computer access activities. Employing a group of therapists and assistive technology professionals to evaluate and demonstrate SITbench would be also quite useful.

## Figures and Tables

**Table 1:** Modified statements of the SUS questionnaire with average scale values of all participants.

Statements	Scale
(1) I would use SITbench for SIT evaluation tasks frequently	4.00
(2) I found SITbench unnecessarily complex	1.37
(3) I found SITbench easy to use	4.12
(4) I would need the support of a technical person to be able to use SITbench	1.75
(5) I found the various functions in SITbench were well integrated	4.37
(6) I thought there was too much inconsistency in SITbench	1.37
(7) I would imagine that most people would learn to use SITbench very quickly	4.25
(8) I found SITbench very cumbersome/awkward to use	1.50
(9) I felt very confident using SITbench	4.12
(10) I need to learn a lot of things before I can use this system	1.25

## Data Availability

The data used to support the findings of this study are available from the corresponding author upon request.
