# Erratum to “SITbench 1.0: A Novel Switch-Based Interaction Technique Benchmark”

**DOI:** 10.1155/2019/2304970

**Published:** 2019-11-27

**Authors:** Cagdas Esiyok, Sahin Albayrak

**Affiliations:** Distributed Artificial Intelligence Laboratory, Technische Universität Berlin, Berlin, Germany

In the article titled “SITbench 1.0: A Novel Switch-Based Interaction Technique Benchmark” [1], there was a structural error in the fifth sentence of the Abstract, where the phrase “On the contrary” should be written as “On the other hand.” Therefore, the fifth sentence should be updated as follows:

“On the other hand, a benchmark application is also required to make performance evaluation of SITs by using a number of standard tests and empirical attributes.”
